# Factors Influencing Patient Decisions Regarding Treatments for Skin Growths: A Cross-Sectional Study

**DOI:** 10.1155/2018/3941347

**Published:** 2018-06-19

**Authors:** David G. Li, Fan Di Xia, Jasmine Rana, Grace J. Young, Forootan Alizadeh, Cara Joyce, Shinjita Das, Arash Mostaghimi

**Affiliations:** ^1^Department of Dermatology, Brigham & Women's Hospital, Harvard Medical School, Boston, MA, USA; ^2^Tufts University School of Medicine, Boston, MA, USA; ^3^Brigham & Women's Hospital, Harvard Medical School, Boston, MA, USA; ^4^Santa Clara Valley Medical Center, San Jose, CA, USA; ^5^Harvard Medical School, Boston, MA, USA; ^6^Loyola University, Chicago, IL, USA; ^7^Department of Dermatology, Massachusetts General Hospital, Harvard Medical School, Boston, MA, USA

## Abstract

Variations in treatment modalities for skin growths contribute substantially to overall healthcare spending within dermatology. However, little is known regarding factors impacting patient decision-making when choosing a treatment modality. In this survey-based, cross-sectional study (n = 375, 81.9% response rate), we asked patients to rate the importance of different treatment parameters for a nonfacial skin growth, further classified into five domains: efficacy, appearance, financial impact, visit duration, and productivity. Although patients generally prioritized treatment efficacy when selecting a treatment modality, they emphasized different aspects of the treatment experience as a function of age, gender, race, insurance status, and history of malignancy. Patients over age 50 were less likely to consider treatment impact on finances as being “important”, but more so efficacy and visit duration. Women were more likely to value efficacy and appearance. Patients without private insurance were more likely to cite efficacy and impact on productivity as being “important”. While the underlying reasons for these variations differ across patients, these findings help explain variations in treatment selection among patients choosing between treatments for skin growths and may ultimately lead to improved shared decision-making.

## 1. Introduction

Skin growths are a common presenting complaint in the outpatient dermatology setting, commonly manifesting as seborrheic keratoses, cysts, warts, lipomas, actinic keratoses, nonmelanoma skin cancers, benign nevi, and malignant melanomas [1, 2]. For each type of skin growth, existing treatment modalities confer different benefits and risks, necessitating individualized patient decision-making when selecting a treatment [3, 4].

Understanding patient characteristics associated with treatment preferences for skin growths may help promote shared decision-making to enhance patient experience and outcomes [5, 6]. Although variations in treatment modalities for skin growths contribute substantially to overall healthcare spending within dermatology, little is known about factors influencing patient decision-making when selecting a treatment modality [7]. In this cross-sectional study, we examined the factors underlying patient decision-making for treatment of skin growths.

## 2. Materials and Methods

We surveyed all patients aged ≥18 years at Brigham and Women's Hospital Dermatology over 5 days in August 2016. Patients were not required to have a history of skin conditions and participation was optional and uncompensated. Study staff provided a survey asking each patient to rate the importance of different treatment parameters for a nonfacial skin growth on a Likert scale between 1 and 5, with responses of 4 or 5 being categorized as “important”. Treatment parameters were subsequently classified into five domains by study staff (DGL, AM): efficacy, appearance, financial impact, visit duration, and productivity (Supplemental Materials, available here). In addition, respondents were also asked to provide information on age, gender, insurance coverage, number of dermatology visits, number of biopsies in the past 5 years, and history of skin cancer.

Clinical and demographic information were reported descriptively using means (standard deviation) and percentages (Table 1). Percentage of respondents who rated each variable as 4 or 5 was calculated. Multivariable logistic regression analyses of patient characteristics associated with decision domains were performed. Statistical analyses were performed using SAS 9.4 (SAS Institute).

## 3. Results

458 surveys were administered, of which 375 surveys (81.9% response rate) were completed. Treatment efficacy was considered an important factor by most (68.5%, n = 243) and visit duration (33.1%, n = 118) by the fewest. Patients over age 50 were less likely than those younger than 50 to consider treatment impact on finances (odds ratio [OR] 0.47 [95% CI 0.28-0.78]) as being “important”, but more likely to consider efficacy (OR 1.78 [1.03-3.05]) and visit duration (OR 2.16 [1.26-3.71]) (Table 2). Women were twice as likely as men to value efficacy (OR 2.07 [1.27-3.36]) and appearance (OR 1.98 [1.23-3.19]). Non-white patients more frequently valued financial impact (OR 2.80 [1.49-5.29]) and visit duration (OR 2.60 [1.41-4.78]) than did white patients. Patients without private insurance were more likely than those with private insurance to cite efficacy (OR 2.11 [1.20-3.68]) and impact on productivity (OR 2.24 [1.35-3.71]) as being “important”. Patients without a history of skin cancer emphasized appearance (OR 2.71 [1.56-4.73]), financial impact (OR 1.92 [1.11-3.32]), and visit duration (OR 2.34 [1.33-4.14]) over those with skin cancer history.

## 4. Discussion

This study highlights differences in prioritization among patients when deciding how to treat skin growths. Although patients overall prioritize treatment efficacy when making decisions, they emphasize different aspects of the treatment experience as a function of age, gender, race, insurance status, and history of malignancy.

While the underlying reasons for these variations differ across patients, many of these findings are consistent with known preferences. Patients over 50 are more likely to have a malignant skin growth compared to younger patients, thus being more likely to value treatment efficacy than their younger counterparts. Women's emphasis on appearance is consistent with greater use of plastic surgery cosmetic procedures, 92% of which are performed on female patients [8]. Non-white patients have been reported to earn less than their white counterparts, which may explain the greater emphasis on financial impact of treatment among non-white patients [9, 10]. Additionally, patients without private insurance may prefer treatment options minimizing the impact on productivity, as these patients may be more likely to be of lower socioeconomic status, thus necessitating an earlier return to work [11, 12].

These findings help explain variations in treatment choice among patients choosing between treatments that may have different treatment experiences and costs but similar clinical outcomes [13]. Although these differences impact treatment choice, they are overlooked in bundled payment models and may place patient preferences at odds with physician reimbursement [14]. While these results are specific to the treatment of skin growths in dermatology, these principles are applicable to medicine broadly, whenever patients have to choose between treatments with comparable clinical outcomes but differences in patient experience. These findings may therefore be broadly informative to patients and clinicians in explaining variations in treatment choices and support current efforts to use patient reported outcomes to capture more completely factors that influence patient decision-making.

Although this study contains a large sample size, our findings must be interpreted in the context of the study design. This study was conducted in a single academic medical center and study findings may not be generalizable to other patient populations. Additionally, because the survey does not specify the malignancy status associated with the hypothetical skin growth, respondents were free to assume the malignancy status, which may result in nondifferential misclassification bias owing to variable assumptions among survey respondents. Finally, although the study is cross-sectional in nature and survey-based, these results are unlikely to be subject to response bias given the high response rate (81.9%) among survey respondents.

## 5. Conclusion

Although treatment modalities for skin growths contribute considerably toward spending within dermatology, clinician understanding regarding factors impacting treatment selection is limited. These study findings are a step toward explaining variations in treatment selection among patients choosing between treatments for skin growths. Replication of these findings and a closer consideration of patient preferences across other spheres of care may help explain variations in practice.

## Figures and Tables

**Table 1:** Participant characteristics.

	Overall n = 375
Age, mean (SD)	51.4 (18.9)

Age ≤ 35	101 (26.9)

Female, n (%)	229 (61.1)

Race, n (%)	

White	306 (83.2)

Hispanic	25 (6.8)

African American	19 (5.2)

Asian	12 (3.3)

Other	6 (1.6)

Insurance, n (%)	

Private	231 (61.6)

Medicare	93 (24.8)

Medicaid	46 (12.3)

Self-insured/self-pay	4 (1.1)

Other/unknown	1 (0.3)

Dermatology visits in past 5 years, n (%)	

0-2	79 (21.1)

3-5	105 (28.0)

>5	191 (50.9)

Skin biopsies in past 5 years, n (%)	

0-2	329 (87.7)

3-5	35 (9.3)

>5	11 (2.9)

History of melanoma	42 (11.2)

History of SCC/BCC	98 (26.1)

**Table 2:** Adjusted odds ratios for patient characteristics associated with preferences about treatment approaches of skin growths.

	**Treatment efficacy**	**Appearance**	**Financial impact**	**Visit Duration**	**Productivity**
n responded	355	347	350	356	353

n (%) important	243 (68.5)	144 (41.5)	148 (42.3)	118 (33.1)	133 (37.7)

	**Odds ratio (95% Confidence Interval)**				

Age					

≤50	1 (reference)	1 (reference)	1 (reference)	1 (reference)	1 (reference)

>50	1.78 (1.03, 3.05)†	1.22 (0.72, 2.05)	0.47 (0.28, 0.78)‡	2.16 (1.26, 3.71)‡	0.84 (0.50, 1.43)

Gender					

Male	1 (reference)	1 (reference)	1 (reference)	1 (reference)	1 (reference)

Female	2.07 (1.27, 3.36)‡	1.98 (1.23, 3.19)‡	1.18 (0.74, 1.9)	1.06 (0.65, 1.72)	1.50 (0.93, 2.40)

Race					

White	1 (reference)	1 (reference)	1 (reference)	1 (reference)	1 (reference)

Non-White	1.77 (0.88, 3.56)	1.82 (0.98, 3.36)	2.80 (1.49, 5.29)‡	2.60 (1.41, 4.78)‡	1.67 (0.91, 3.04)

Insurance					

Private	1 (reference)	1 (reference)	1 (reference)	1 (reference)	1 (reference)

Public/self-pay	2.11 (1.20, 3.68)‡	0.91 (0.54, 1.51)	1.07 (0.64, 1.81)	1.37 (0.82, 2.28)	2.24 (1.35, 3.71)‡

History of skin cancer					

Yes	1 (reference)	1 (reference)	1 (reference)	1 (reference)	1 (reference)

No	1.03 (0.59, 1.80)	2.71 (1.56, 4.73)‡	1.92 (1.11, 3.32)†	2.34 (1.33, 4.14)‡	1.32 (0.77, 2.26)

†p < 0.05, ‡p < 0.01

## Data Availability

All data is available in Tables 1 and 2, and within the supplementary materials.

## Additional Points

*Reprint Requests*. Arash Mostaghimi, MD, MPA, MPH.

## Supplementary Materials

Supplementary Materials (1) “ISCEP Survey: Skin Growths”: (a) survey evaluating different domains of patient decision-making for the treatment of a nonspecific skin growth. (2) “Classification of Domains”: (a) classification scheme identifying decision-making domains for survey questions.
