# Supplementary material for: Factors Influencing Patient Decisions Regarding Treatments for Skin Growths: A Cross-Sectional Study
Source: Dermatol Res Pract. 2018 Jun 19;2018:3941347. doi: 10.1155/2018/3941347 (PMC6029444; doi:10.1155/2018/3941347)
Supplement: Supplementary Materials — (1) “ISCEP Survey: Skin Growths”: (a) survey evaluating different domains of patient decision-making for the treatment of a nonspecific skin growth. (2) “Classification of Domains”: (a) classification scheme identifying decision-making domains for survey questions. [file 3941347.f1.docx]

| **ISCEP Survey: Skin Growths**  **Please circle how important each factor is to you when choosing a treatment for a skin growth on an area of skin that is NOT YOUR FACE.**   \|  \| ***Not at all important*** \| \|  \|  \|  \| ***Very important*** \| \|  \| \| --- \| --- \| --- \| --- \| --- \| --- \| --- \| --- \| --- \| \| How long it takes to be performed (in clinic or at home) \| \| 1 \| 2 \| 3 \| 4 \| \| 5 \|  \| \| How much it costs \| \| 1 \| 2 \| 3 \| 4 \| \| 5 \|  \| \| How much it hurts \| \| 1 \| 2 \| 3 \| 4 \| \| 5 \|  \| \| Being able to perform it myself at home \| \| 1 \| 2 \| 3 \| 4 \| \| 5 \|  \| \| Having my dermatologist perform it in clinic \| \| 1 \| 2 \| 3 \| 4 \| \| 5 \|  \| \| How long I have to take care of the treated area after treatment \| \| 1 \| 2 \| 3 \| 4 \| \| 5 \|  \| \| Temporary activity restrictions after treatment, such as avoiding sun, exercise or heavy lifting \| \| 1 \| 2 \| 3 \| 4 \| \| 5 \|  \| \| Having to return for a follow-up appointment (to see if the treatment worked or for wound care) \| \| 1 \| 2 \| 3 \| 4 \| \| 5 \|  \| \| If possible, microscopic confirmation that the treatment was successful (e.g. the growth was completely removed) \| \| 1 \| 2 \| 3 \| 4 \| \| 5 \|  \| \| Risk the skin growth may come back in the future and need additional treatment \| \| 1 \| 2 \| 3 \| 4 \| \| 5 \|  \| \| Risk of skin discoloration after treatment \| \| 1 \| 2 \| 3 \| 4 \| \| 5 \|  \| \| Risk of scarring after treatment or how the scar looks (if present) \| \| 1 \| 2 \| 3 \| 4 \| \| 5 \|  \| |  |
| --- | --- | --- | --- | --- | --- | --- | --- | --- | --- | --- | --- | --- | --- | --- | --- | --- | --- | --- | --- | --- | --- | --- | --- | --- | --- | --- | --- | --- | --- | --- | --- | --- | --- | --- | --- | --- | --- | --- | --- | --- | --- | --- | --- | --- | --- | --- | --- | --- | --- | --- | --- | --- | --- | --- | --- | --- | --- | --- | --- | --- | --- | --- | --- | --- | --- | --- | --- | --- | --- | --- | --- | --- | --- | --- | --- | --- | --- | --- | --- | --- | --- | --- | --- | --- | --- | --- | --- | --- | --- | --- | --- | --- | --- | --- | --- | --- | --- | --- | --- | --- | --- | --- | --- | --- | --- | --- | --- | --- | --- | --- | --- | --- | --- | --- | --- | --- | --- | --- |

**Classification of Domains:**

The below questions were averaged within domain, and an average ≥ 4 was considered “important”

**Treatment Efficacy**

- Risk the skin growth may come back and need additional treatment
- If possible, microscopic confirmation that the treatment was successful
- Having my dermatologist perform it in clinic

**Appearance**

- Risk of scarring after treatment or how the scar looks
- Risk of skin discoloration after treatment

**Financial impact**

- How much it costs

**Visit Duration**

- How long it takes to be performed
- Having to return for a follow-up appointment

**Productivity**

- How long I have to take care of the treated area after treatment
- Temporary activity restrictions after treatment
- Risk the skin growth may come back and need additional treatment
- If possible, microscopic confirmation that the treatment was successful
- Having my dermatologist perform it in clinic
